# A systematic review and meta-analysis of the effect of high-intensity statin on coronary microvascular dysfunction

**DOI:** 10.1186/s12872-023-03402-9

**Published:** 2023-07-24

**Authors:** Bihan Huang, Xueying Han, Yun Pan, Dongdong Chen

**Affiliations:** 1 Department of Cardiology, Huazhong University of Science and Technology Union Shenzhen Hospital, Shenzhen, China; 2 Department of Cardiology, The First Affiliated Hospital of Jinan University, Guangzhou, China

**Keywords:** High-intensity statin, Thrombolysis in myocardial infarction (TIMI), Myocardial blush grade (MBG), Index of microvascular resistance (IMR), Meta-analysis

## Abstract

**Objective:**

The purpose of this meta-analysis is to evaluate the role of high-intensity statin pretreatment on coronary microvascular dysfunction in patients with coronary heart disease undergoing percutaneous coronary intervention (PCI).

**Methods:**

PubMed, Cochrane, and Embase were searched. This meta-analysis selection included randomized controlled trials (RCTs), involving high-intensity statin pretreatment as active treatment, and measurement of thrombolysis in myocardial infarction (TIMI), myocardial blush grade (MBG) or index of microvascular resistance (IMR) in coronary heart disease (CHD) patients undergoing PCI. I^2^ test was used to evaluate heterogeneity. Pooled effects of continuous variables were reported as Standard mean difference (SMD) and 95% confidence intervals (CI). Pooled effects of discontinuous variables were reported as risk ratios (RR) and 95% confidence intervals (CI). Random-effect or fix-effect meta-analyses were performed. The Benefit was further examined based on clinical characteristics including diagnosis and statin type by using subgroup analyses. Publication bias was examined by quantitative Egger’s test and funnel plot. We performed sensitivity analyses to examine the robustness of pooled effects.

**Results:**

Twenty RCTs were enrolled. The data on TIMI < 3 was reported in 18 studies. Comparing with non-high-intensity statin, high-intensity statin pretreatment significantly improved TIMI after PCI (RR = 0.62, 95%CI: 0.50 to 0.78, *P* < 0.0001). The data on MBG < 2 was reported in 3 studies. The rate of MBG < 2 was not different between groups (RR = 1.29, 95% CI: 0.87 to 1.93, *P* = 0.21). The data on IMR was reported in 2 studies. High-dose statin pretreatment significantly improved IMR after PCI comparing with non-high-dose statin (SMD = -0.94, 95% CI: -1.47 to -0.42, *P* = 0.0004). There were no significant between-subgroup differences in subgroups based on statin type and diagnosis. Publication bias was not indicated by using quantitative Egger’s test (*P* = 0.97) and funnel plot. Sensitivity analyses confirmed the robustness of these findings.

**Conclusions:**

Comparing with non-high-intensity statin, high-intensity statin pretreatment significantly improved TIMI and IMR after PCI. In the future, RCTs with high quality and large samples are needed to test these endpoints.

**Supplementary Information:**

The online version contains supplementary material available at 10.1186/s12872-023-03402-9.

## Introduction

Percutaneous coronary intervention (PCI) has been widely used in revascularization therapy for patients with coronary heart disease, especially for acute coronary syndrome (ACS). Early removal of coronary artery stenosis or occlusion and recovery of coronary blood flow play an important role in alleviating patients' symptoms and reducing mortality, which has been unanimously recommended by national guidelines [1, 2]. However, because of Coronary microcirculation dysfunction (Coronary microvascular dysfunction, CMD), there is still a larger proportion of patients with successful opening of the narrowed or occluded epicardial coronary arteries without recovery of blood flow in the distal coronary microvessels, myocardial perfusion is not truly effective, leading to no significant relief of symptoms and increasing the risk of cardiovascular adverse events in patients with CHD [3, 4]. The pathophysiology of CMD is very complex, involving endothelial dysfunction, platelet activation, microvascular thromboembolism, and other mechanisms. How to improve the CMD after PCI treatment in patients with coronary heart disease has been paid high attention by clinical workers.

Statins are the cornerstone of drug therapy for coronary heart disease, and are competitive inhibitors of 3-hydroxy-3-methylglutaryl-coenzyme A reductase (HMG-CoA). It can not only inhibit the synthesis of cholesterol but also reduce the cholesterol level by increasing the low-density lipoprotein (LDL) receptor on liver cells and strengthening the endocytosis of LDL receptor-mediated [4]. In addition to the strong cholesterol-lowering effect, statins also have a variety of effects including platelet inhibition, vasodilation, inflammation inhibition, and improvement of endothelial function [5–8], which contribute to the improvement of microcirculation function [9].

At present, the effects of statin with different dose intensity on the improvement of coronary microcirculation in patients with CHD after PCI have been studied, but the results are controversial. Studies have reported that high-intensity statin before PCI can significantly improve the dysfunction of coronary microcirculation after PCI compared with low-dose statin [10]. However, other studies have shown that high-intensity statins do not improve CMD in patients with CHD after PCI compared with low-dose statins [11, 12]. The differences in research results may be related to the design factors. Therefore, the purpose of this study was to perform a meta- analysis based on the collection of relevant RCTs to evaluate whether high-intensity statins are more effective in improving coronary microcirculation in patients with CHD after PCI than the low-dose intensive statins.

## Methods

The meta-analysis was conducted with conforming to Preferred Reporting Items for Systematic Review and Meta-analyses (PRISMA) guidelines. (Registration in PROSPERO: CRD42020184732) (Additional file 1: Table S1).

### Search strategy

A comprehensive search of PubMed, Cochrane, and Embase databases was conducted for all published papers comparing the effects of high-intensity and non-high-intensity statin pretreatment statin on microcirculatory function in patients with CHD after PCI, without restriction of language. The retrieval time is up to March, 2020. Retrieval keywords include Statin, Percutaneous intervention, and Randomized Controlled trial. The literature types were all RCT. This study also carried out manual retrieval of the references of relevant papers.

### Inclusion and exclusion criteria

#### Inclusion criteria

Population: patients with CHD, including ACS and stable angina.

Treatment measures of high-intensity statin group: High-intensity statins were given before PCI. As we know, high-intensity statins were defined as atorvastatin 40-80 mg/day, Rosuvastatin 20-40 mg/day, and simvastatin 80 mg/day [13].

Treatment measures of non-high-intensity statin group: Low dose, no statins or placebo were administered before PCI.

The endpoints: Related indexes of coronary microcirculation after PCI such as Thrombolysis in myocardial infarction(TIMI)、Myocardial blush grade(MBG) and index of microvascular resistance(IMR) [14].

The type of study: Peer-reviewed RCT studies.

#### Exclusion criteria

(1)Non-RCTs, (2)head-to-head comparisons of different statins, (3)studies without PCI as a part of the protocol, (4)studies with revascularization as an exclusion criterion, (5)unavailable data on CMD, and (6)duplicate articles were excluded.

### Study selection and data extraction

The literature was screened separately by two authors. In case of any disagreement, discuss with the third author to reach a consensus on inclusion. According to the inclusion and exclusion criteria, the two authors initially selected the relevant articles by reading the titles and abstracts respectively. After reading the full text of the preliminarily selected articles and clarifying the included articles, the basic data of each study and relevant endpoints of the study were collected, including the first author's last name, research published time, countries, the number of population, diagnosis, the patient's age, sex ratio, statin type, and dosage, studies the endpoints, etc.

### Quality assessment

The two authors used the Cochrane Collaboration Risk Assessment tool to evaluate the quality of the included studies [15], and if there were differences in the evaluation process, they discussed with the third author to reach consensus. Specific quality assessment items include the following kinds of bias risk assessment: risk of selective bias, risk of implementation bias, risk of measurement bias, risk of follow-up bias, risk of reporting bias, and other bias risks. For each biased item, low risk, high risk, and ambiguity were used for evaluation.

### Statistical analyses

The meta-analysis used STATA 15.1 (STATA Corp. College Station, TX, USA) and Revman 5.3 (Nordic Cochrane Centre, Denmark) for statistical analysis. I^2^ test was used to evaluate heterogeneity, when I^2^ ≤ 50%, it indicated good homogeneity among various tests, and fixed effect model was used for analysis. If I^2^ > 50% indicates heterogeneity among trials, random-effects model was used for analysis. Pooled effects of continuous variables were reported as Standard mean difference (SMD) and 95% CI. Pooled effects of discontinuous variables were reported as risk ratios (RR) and 95%CI. Statistical significance was accepted at *p* < 0.05. Publication bias was examined by quantitative Egger’s test and funnel plot when the number of included studies reaches 10 or more [16]. Benefit was further examined based on clinical characteristics including diagnosis and statin type by using subgroup analyses. Statistically significant subgroup effect was accepted at *p* < 0.01 [17]. Finally, we performed sensitivity analyses to examine the robustness of pooled effects.

## Results

### Literature screening results

**Fig. 1:**
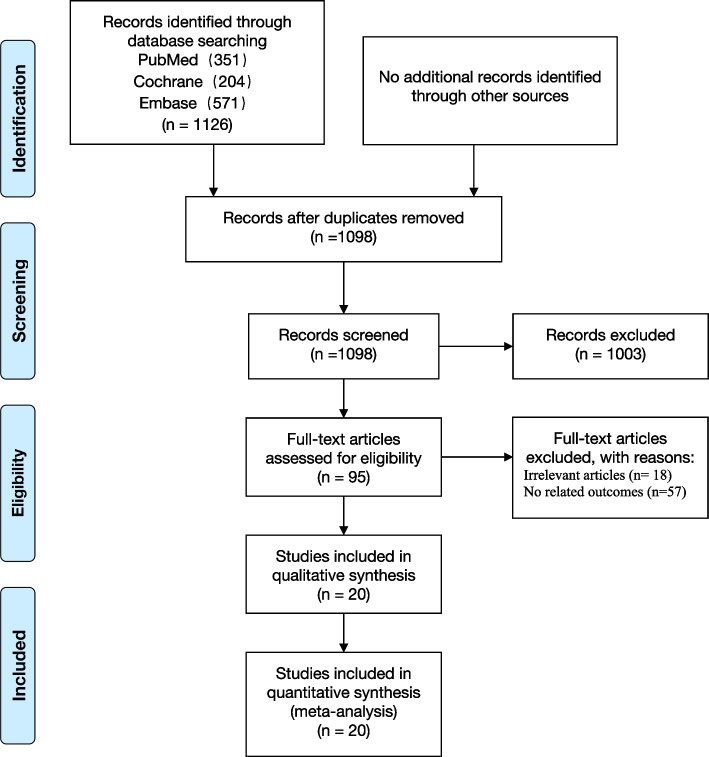
Flow chart for inclusion and exclusion of studies

A total of 1126 related articles were retrieved from PubMed, Cochrane and Embase databases, and 28 duplicate articles were excluded. The remaining 1098 articles were first read by title and abstract, and then the full text of potentially related articles was read to determine whether the inclusion criteria were fit, and finally, 20 RCTS were included [10–12, 18–33]. The specific inclusion process is shown in Fig. 1.

### Characteristics and quality assessment of included studies

A total of 3165 patients were included in the study, including 1535 in the high-intensity statin group and 1630 in the control group. Eighteen studies reported postoperative TIMI, three reported postoperative MBG, and two reported postoperative IMR. In terms of population inclusion, 11 studies were included in the diagnosis of ST-elevation myocardial infarction (STEMI), 2 studies were included in the diagnosis of stable coronary artery disease (SCAD), 2 studies were included in the diagnosis of unstable angina (UA), only 1 study was included in the diagnosis of coronary artery disease (CAD), 1 study was included in the diagnosis of non-ST-elevated myocardial infarction (NSTEMI), 2 studies were included in the diagnosis of non-ST-elevation acute coronary syndrome(NSTE-ACS), only 1 study was included in the diagnosis of ACS. And in terms of statins, there were 17 studies using atorvastatin, 2 studies using rosuvastatin, and only 1 study using simvastatin. The study characteristics and patient characteristics of the 20 studies are shown in Tables 1 and 2.

**Table 1:** Features of studies included in this meta-analysis

Research	Year	country	Sample size	Diagnosis	Type	History	Dosage regimen (statin / contrast)	Endpoints
Jia	2009	China	228	ACS	Simvastatin	NA	80 mg/20 mg	TIMI
STATIN STEMI	2009	Korea	171	STEMI	Atorvastatin	statin naïve	80 mg/10 mg	TIMI
Hahn	2011	Korea	173	STEMI	Atorvastatin	statin naïve	80 mg/no statin	TIMI MBG
REPERATOR	2012	Netherlands	42	STEMI	Atorvastatin	statin naïve	80 mg/placebo	TIMI
Chen	2013	China	156	STEMI	Atorvastatin	statin naïve	80 mg 1.5 h before/placebo 1.5 h before angiography	TIMI
He	2013	China	84	SCAD	Atorvastatin	NA	40 mg/10 mg	IMR
Liu	2013	China	102	STEMI	Atorvastatin	NA	80 mg/no statin	TIMI
Takano	2013	Japan	210	SCAD	Rosuvastatin	statin naïve	20 mg from 5 to 7 days before planned intervention/2.5 mg	TIMI
ROSEMARY	2014	Korea	132	STEMI	Rosuvastatin	NA	80 mg/placebo	TIMI
Su	2014	China	66	UA	Atorvastatin	statin naïve	80 mg/20 mg	TIMI
AT-STEMI	2015	Korea	67	STEMI	Atorvastatin	statin naïve	80 mg/no statin	TIMI、MBG
SAMIT	2015	Japan	190	STEMI	Atorvastatin	NA	40 mg vs. no statin	TIMI
Shehata	2015	Egypt	118	NSTE-ACS	Atorvastatin	NA	80 mg 12 and 2 h before angiography/no statin	TIMI
Yang	2015	China	96	UA	Atorvastatin	statin naïve	80 mg/20 mg	TIMI
Liu	2016	China	798	CAD	Atorvastatin	NA	80 mg/no statin	TIMI
RESIST-ACS	2016	Korea	77	NSTE-ACS	Atorvastatin	statin naïve	80 mg within 12 to 24 h and 40 mg 2 h before PCI vs.10 mg 12 to 24 h before PCI	IMR
Yan	2016	China	114	STEMI	Atorvastatin	NA	80 mg/20 mg	TIMI
Liu	2017	China	138	STEMI	Atorvastatin	NA	40 mg vs. 20 mg or no statin	TIMI
Shehata	2017	Egypt	100	NSTEMI	Atorvastatin	statin naïve	80 mg 24 and 12 h before angiography/no statin	TIMI
García-Méndez	2018	Mexico	103	STEMI	Atorvastatin	NA	80 mg/no statin	TIMI、MBG

*NA* No data available, *ACS* acute coronary syndrome, *STEMI* ST-segment elevation myocardial infarction, *Nste-ACS* Non-ST-segment elevation acute coronary syndrome, *TIMI* thrombolysis in myocardial infarction, *MBG* Myocardial blush grade, *IMR* index of microvascular resistance

**Table 2:** Demographic and baseline characteristics of studies included in this meta-analysis

Research	year	Age (statin /contrast %)	Male (statin /contrast, %)	Diabetes (statin /contrast, %)	Hypertension (statin / contrast, %)	Dyslipidemia (statin / contrast, %)
Jia	2009	65.4 ± 10.9/65.7 ± 12.1	69.9/58.3	19.5/21.7	65.5/61.7	NA
STATIN STEMI	2009	61 ± 11/59 ± 11	76.7/77.6	24.5/18.9	52.3/46.4	NA
Hahn	2011	55.5 ± 12.1/59.7 ± 12.8	85.4/82.1	28.1/21.4	43.8/46.4	50.6/51.2
REPERATOR	2012	57.5 ± 7.7/64.6 ± 10.3	65/86	5/23	55/23	NA
Chen	2013	60.71 ± 12.4/61.83 ± 12.21	72.4/67.5	28.9/31.3	60.5/68.8	NA
He	2013	66.8 ± 9.6/63.6 ± 10.4	74.4/68.3	25.6/34.1	65.1/61.1	NA
Liu	2013	59.3 ± 9.96/62.1 ± 11.4	81.25/78.57	12.5/10	68.75/48.57	25/31.43
Takano	2013	69 ± 10/68 ± 9	76.4/77.9	50/51.9	76.9/71.7	NA
ROSEMARY	2014	57.7 ± 12.0/57.21 ± 11.0	86/86	27/21	61/37	NA
Su	2014	65.54 ± 9.35/65.57 ± 11.45	85/82	27/21	61/58	27/33
AT-STEMI	2015	57.4 ± 10.7/59.1 ± 13.3	93.3/91.9	23.3/18.9	33.3/37.8	60/43.2
SAMIT	2015	61 ± 13/62 ± 12	82/75	20/23	50/49	36/26
Shehata	2015	57 ± 8/58 ± 9	67/68	52/47	55/57	38/40
Yang	2015	65.4 ± 9.7/65.8 ± 11.5	83/79	33/29	63/63	50/46
Liu	2016	61.8 ± 10.1/62.5 ± 11.2	73.3/71.4	31.8/32.7	65/64.1	NA
RESIST-ACS	2016	66.1 ± 9.3/67.7 ± 8.2	76.9/68.4	28.2/29	59/65.8	53.9/44.7
Yan	2016	57 ± 13/55 ± 11	81.36/80	35.59/43.64	64.41/58.18	NA
Liu	2017	57.8 ± 6.4/62.05 ± 4.16	43.48/53.26	NA	NA	NA
Shehata	2017	56 ± 9/55 ± 11	66/70	54/50	66/68	44/40
García-Méndez	2018	64 ± 11/64 ± 11	89/85	45/30	53/63	47/39

*NA* No data available

**Fig. 2:**
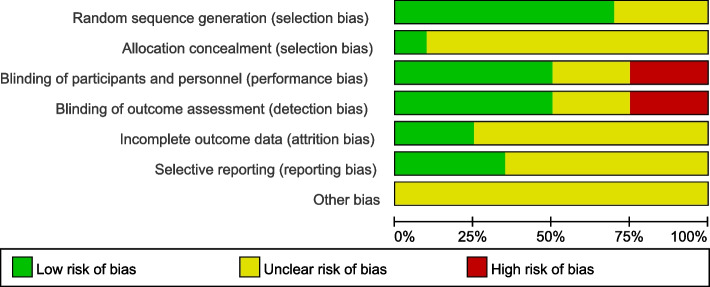
Risk bias graph of the included studies

**Fig. 3:**
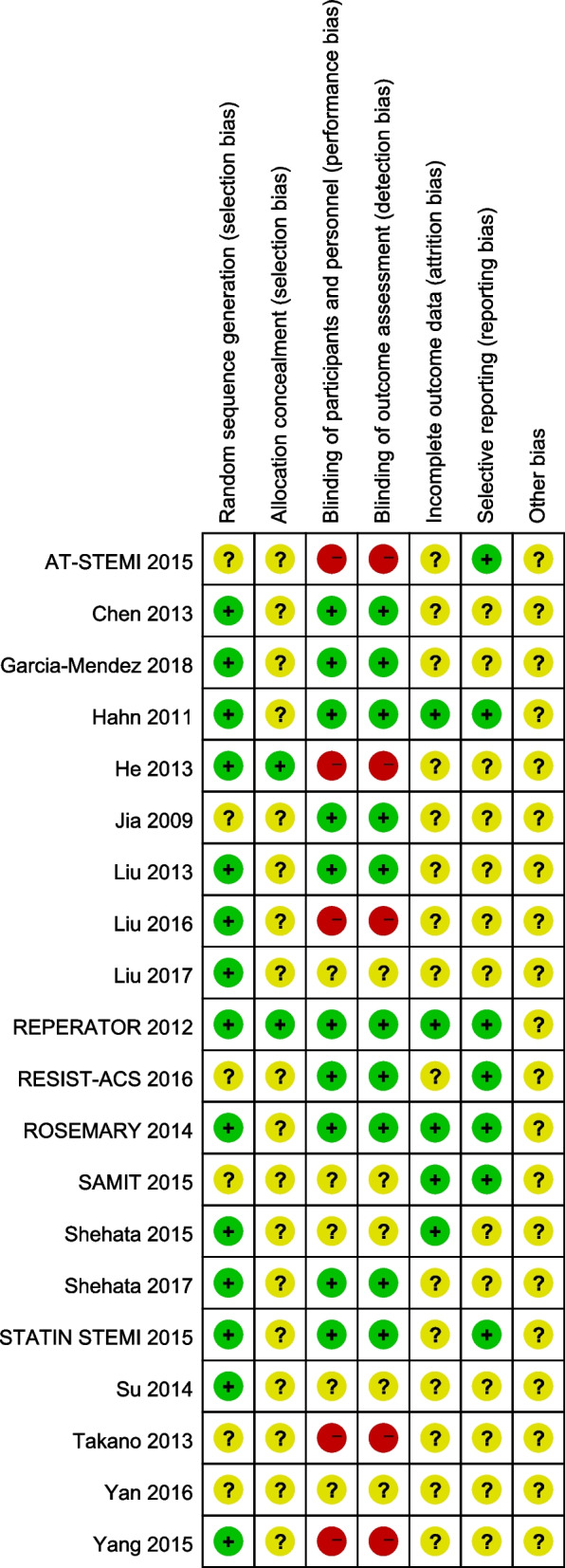
Summary chart of risk bias in the included studies

The risk of bias was assessed in 20 studies. In terms of selection bias, randomization was described in detail and correctly in 14 studies. 2 studies describe the correct method of allocating concealment. As for the blind method, patients and evaluators were blinded in 5 studies, only evaluators were blinded in 5 studies, and patients and evaluators were not blinded in 4 studies. In terms of follow-up bias, 5 studies described the number of missing persons and the causes, which were similar between groups. With regard to reporting bias, seven studies had published research proposals. There was no other risk bias was mentioned in the 20 studies (Figs. 2 and 3).

### Results of the included studies

#### TIMI grading

**Fig. 4:**
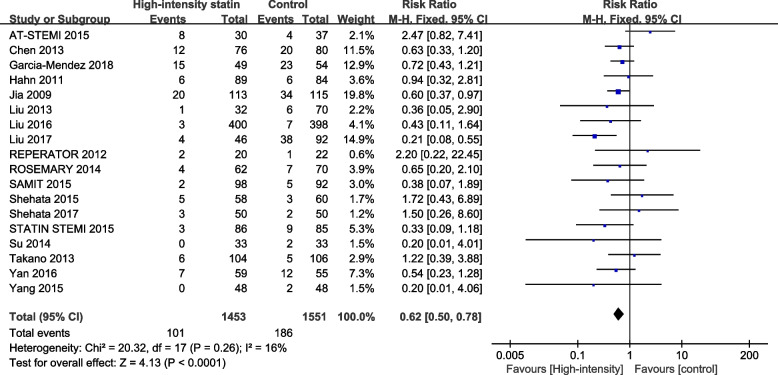
Forest plot for TIMI < 3 between high-intensity statins and control group. M-H = Mantel–Haenszel; RR = risk ratio

Eighteen studies reported postoperative TIMI < 3. Heterogeneity analysis suggested that there was no significant heterogeneity among randomized controlled trials (I^2^ = 16%, *P* = 0.26). The results of fixed-effect model analysis showed that 7.0% of patients (101/1453) in the high-intensity statin treatment group had postoperative TIMI c. In the control group, patients with postoperative TIMI < 3 accounted for 12.0% (186/1551). The results showed that high-intensity statin pretreatment significantly improved postoperative TIMI grading (RR = 0.62, 95%CI: 0.50 to 0.78, *P* < 0.0001) (Fig. 4).

#### MBG grading

**Fig. 5:**
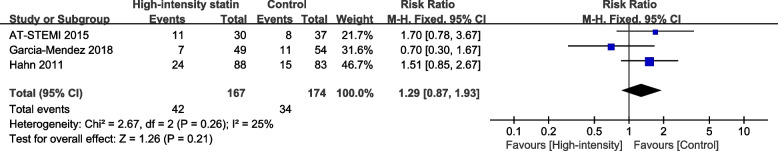
Forest plot for MBG < 2 between high-intensity statins and control group

Three studies reported postoperative MBG < 2. Heterogeneity analysis suggested that there was no significant heterogeneity among randomized controlled trials (I^2^ = 25%, *P* = 0.26). The results of fixed-effect model analysis showed that 25.1% of patients (42/167) in the high-intensity statin group received postoperative MBG < 2, and 19.5% (34/174) in the control group received postoperative MBG < 2. There was no significant effect on the two groups(RR = 1.29, 95% CI: 0.87 to 1.93, *P* = 0.21) (Fig. 5).

#### IMR

**Fig. 6:**
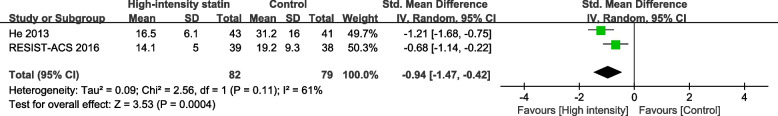
Forest plot for IMR between high-intensity statins and control group

Two studies reported results of IMR immediately after surgery. Heterogeneity analysis suggested significant heterogeneity among randomized controlled trials (I^2^ = 61%, *P* = 0.11). The results of fixed-effect model analysis showed that the high-intensity statin group significantly reduced postoperative IMR compared with the control group (SMD = -0.94, 95% CI: -1.47 to -0.42, *P* = 0.0004) (Fig. 6).

### Subgroup analysis

**Fig. 7:**
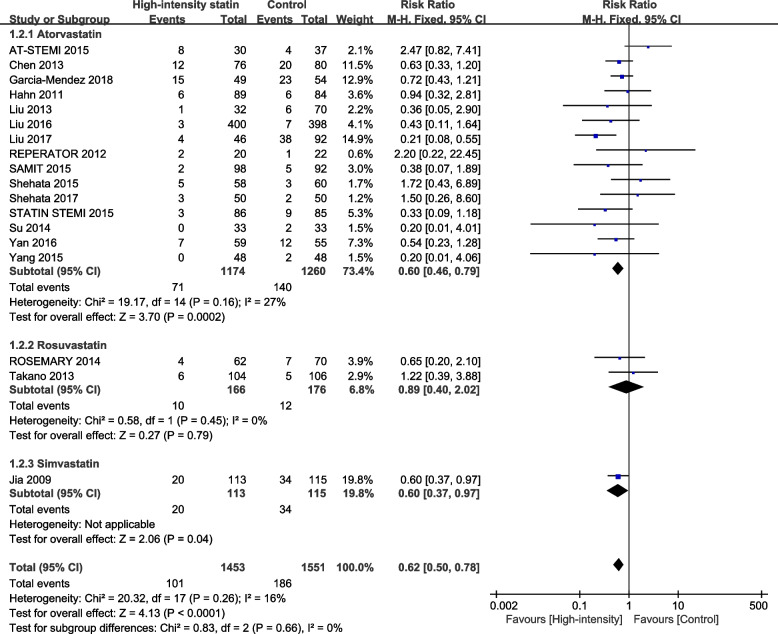
Subgroup analysis of different types of statin in the comparison of high-intensity statin and control group TIMI < 3

**Fig. 8:**
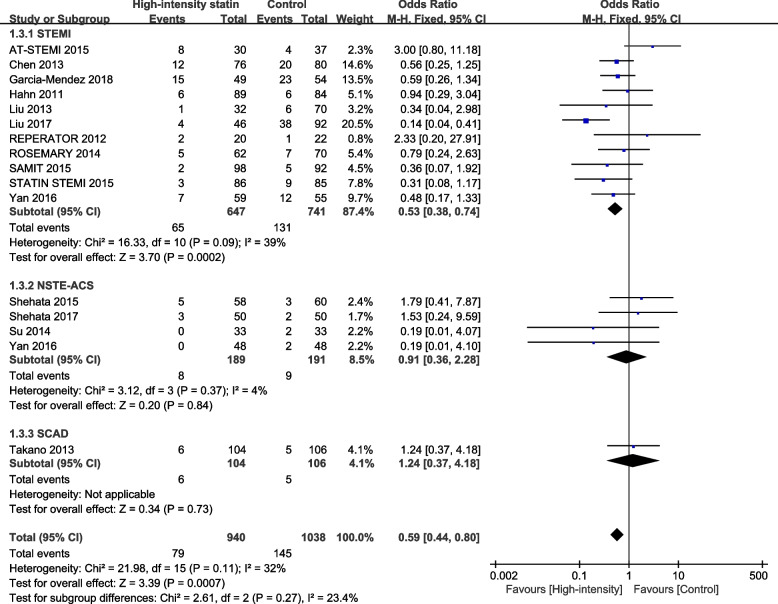
Subgroup analysis of different types of clinical manifestations in the comparison of high-intensity statin and control group TIMI < 3

As mentioned above, subgroup difference test was conducted according to different statin types, and statistical results as shown in Fig. 7 showed that there was no subgroup effect between different statin types (*P* = 0.66). It showed that different statin types did not affect the intervention. According to different clinical manifestations for subgroup difference test, the statistical results (*P* = 0.27) showed that there was no subgroup effect among patients with different clinical manifestations, indicating that there was no significant difference in intervention effects among different clinical manifestations (Fig. 8).

### Publication bias detection

**Fig. 9:**
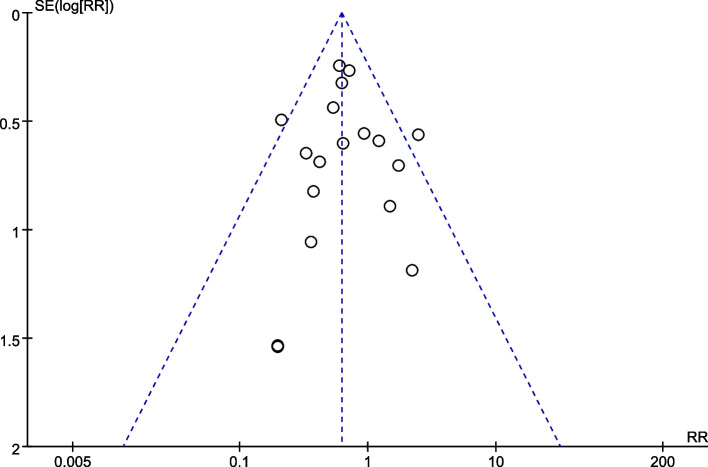
Funnel plots for TIMI < 3 between high-intensity statins and control group

Similarly, funnel plot and Egger check method are used in this study to detect publication bias. As shown in Fig. 9, funnel plot is basically symmetric. In addition, the Egger check results (*P* = 0.97) also indicate that there was no obvious publication bias.

### Sensitivity analysis

**Fig. 10:**
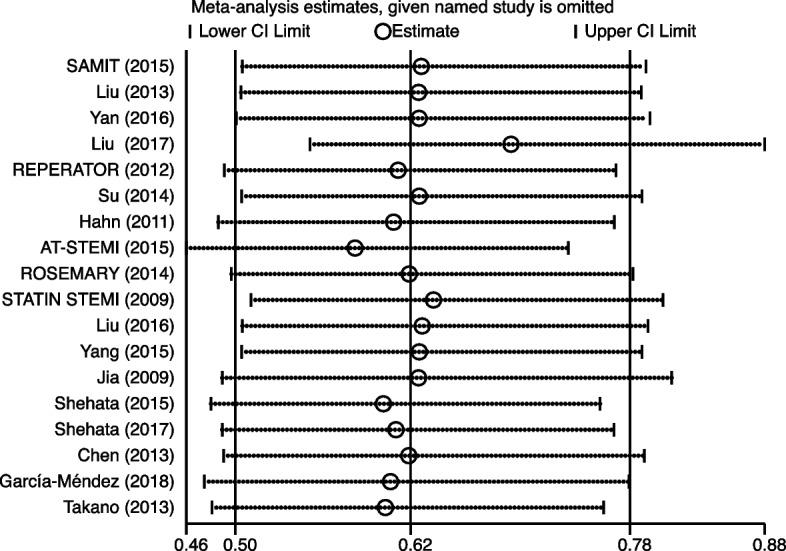
Sensitivity analysis for TIMI between high-intensity statins and control group

**Fig. 11:**
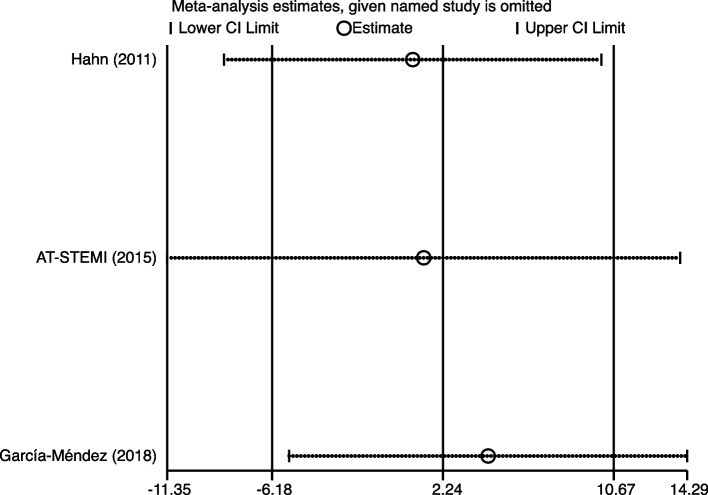
Sensitivity analysis for MBG between high-intensity statins and control group

**Fig. 12:**
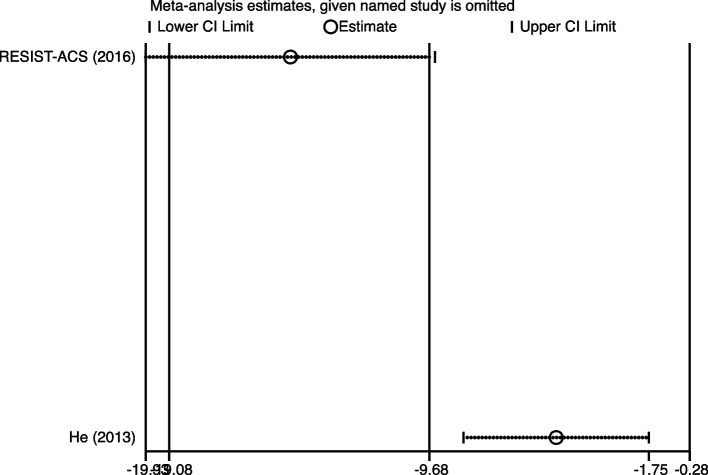
Sensitivity analysis for IMR between high-intensity statins and control group

As shown in Figs. 10, 11 and 12, in the sensitivity analysis of TIMI, MBG, and IMR, none of the trials excluded from the meta-analysis significantly changed the results, indicating the robustness of the results (Figs. 10, 11 and 12).

## Discussion

A total of 20 randomized controlled trials were included in this study. TIMI grading was reported in 18 RCTs, MBG was reported in 3 RCTs, and IMR was reported in 2 RCTs. Firstly, the meta-analysis showed that compared with non-high-intensity statins, high-intensity statins significantly improved TIMI grading and significantly reduced IMR after PCI in patients with CHD. There was no significant difference in postoperative MBG between the two groups. Secondly, subgroup differences were detected for TIMI grading according to clinical diagnosis and statin types in the study, and the results showed that there were no subgroup differences in benefits between different clinical diagnoses and different statin types. Thirdly, sensitivity analysis was conducted in this study, the results showed no significant changes in the meta-analysis results, indicating the robustness of the results. Fourthly, this study detected potential publication bias through funnel plot and quantitative Egger inspection method, and the results showed that there was no publication bias.

Microcirculation is not only an important determinant of effective restoration of myocardial perfusion after PCI to reduce myocardial cell damage and improve cardiac function in patients with CHD, but also an important factor affecting the prognosis of patients [34].

Statins are competitive inhibitors of HMG-CoA reductase [4]. High-intensity statins significantly reduce LDL_C levels, further reducing the risk of ischemic events in patients with CHD. Based on this, national guidelines suggest that patients with CHD should be initiated early with high-intensity statins [1, 2, 35]. In addition to the strong cholesterol-lowering effect, statins also have a variety of effects including platelet inhibition, vasodilation, inflammation inhibition, and improvement of endothelial function [5–8], which contribute to the improvement of microcirculation function [9].

Due to technical reasons, it is still not possible to directly observe the blood flow of coronary microvessels in the human body. Therefore, most of the previous clinical studies on statin's improvement of CMD mainly used TIMI grading to indirectly reflect the situation of coronary microcirculation perfusion. A meta-analysis of 15 randomized controlled trials showed that high-intensity statins significantly improved post-operative TIMI grading (OR = 0.61, 95% CI: 0.46–0.80, *p* = 0.0005) [36]. A total of 20 RCTS were included in this meta-analysis, 18 of which reported TIMI results. The meta- analysis showed that compared with the control group, TIMI grading was significantly higher in the high-intensity statins group after PCI than in the control group (RR = 0.62, 95%CI: 0.50 to 0.78, *P* < 0.0001). This is consistent with previous findings [36], suggesting that high-dose and high-intensity statins are beneficial for improving microcirculatory perfusion. However, TIMI grading is related to the operator's operation and observation experience, which cannot accurately reflect the microcirculation function. Therefore, indicators such as MBG and IMR were included in this study for comprehensive evaluation, which is of more reference value for us to evaluate the effect of high-intensity statin on microcirculation. IMR is a simple and specific index to reflect the function of coronary microcirculation proposed by Fearon and other scholars in recent years. It has a good correlation with the actual microvascular resistance and can be measured by the guide wire with pressure/thermal sensor. It also has the advantages of good repeatability and is not affected by factors such as heart rate and blood pressure. IMR has been shown to have good prognostic value in both ACS and stable angina patients, and is considered to be a reliable indicator for the evaluation of coronary microvascular function [14]. The meta-analysis showed that although MBG results were similar in the two groups after PCI, high-intensity statins significantly reduced IMR(SMD = -0.94, 95% CI: -1.47 to -0.42, *P* < 0.0004). It also indicates that high-intensity statin can better improve the CMD in patients with CHD after PCI compared with low-dose statin.

Current guidelines recommend that patients with CHD should be treated early with high-intensity statins. However, it is not clear when statins will be given. The results showed that compared with non-high-intensity statin treatment, high-intensity statin treatment effectively improved microcirculation dysfunction after PCI, suggesting that high-intensity statin pretreatment can bring additional benefits to patients, and we should actively give high-intensity statin treatment as early as possible before operation in the absence of contraindications.

The meta-analysis has several advantages. First of all, the study focused on comparing the effects of high-intensity statin with non-high-intensity statin treatment on microcirculation function in patients with CHD after PCI. Different from previous studies, TIMI, MBG, and IMR were included in this meta-analysis to evaluate the effects of high-intensity statin pretreatment on coronary microcirculation. Secondly, only RCTs were included in this meta-analysis to minimize the influence of confounding factors on the results and improve the reliability of the results. Finally, subgroup analysis, sensitivity analysis, and publication bias analysis were performed on the results of this study, which all indicated the robustness of the results.

However, the study also has limitations. First, the characteristics of the included population in each study may be different, which may lead to the existence of bias and heterogeneity. Although the subgroup difference analysis of clinical diagnosis and statin type in this study showed consistency of results, while the study could not exclude the influence of other factors on the results, including the age, complications, ethnic groups, different statin dosage before PCI in non-high-intensity group, and so on. Second, some of the experiments included in the study did not use the blind method, and some of the studies did not mention whether to use the blind method in the literature, which may have an impact on the research results. Third, the number of RCTs on IMR and MBG is small. Therefore, higher-quality studies will be needed to verify the results through more reliable and rigorous methods in the future.

## Conclusions

Compared with non-high-intensity statin treatment, high-intensity statin significantly improved TIMI grading and IMR in patients with CHD after PCI, and effectively improved microcirculation dysfunction. More studies with larger samples and higher quality are still needed to verify the results In the future.

## Supplementary Information

**Additional file 1: Table S1.** PRSIMA 2020 Checklist

## Data Availability

All data and material used for this study are available through the corresponding author upon reasonable request.
